# Detection of Local Prostate Cancer Recurrence from PET/CT Scans Using Deep Learning

**DOI:** 10.3390/cancers17091575

**Published:** 2025-05-06

**Authors:** Marko Korb, Hülya Efetürk, Tim Jedamzik, Philipp E. Hartrampf, Aleksander Kosmala, Sebastian E. Serfling, Robin Dirk, Kerstin Michalski, Andreas K. Buck, Rudolf A. Werner, Wiebke Schlötelburg, Markus J. Ankenbrand

**Affiliations:** 1 Center for Computational and Theoretical Biology, Julius-Maximilians-University Würzburg, 97070 Würzburg, Germany; 2 Department of Nuclear Medicine, Dr. Burhan Nalbantoglu State Hospital, Nicosia 99010, Cyprus; huli@hotmail.co.uk; 3 Department of Nuclear Medicine, University Hospital Würzburg, 97080 Würzburg, Germanybuck_a@ukw.de (A.K.B.);; 4 Department of Nuclear Medicine, LMU University Hospital, LMU Munich, 80539 München, Germany; 5 Department of Bioinformatics, Julius-Maximilians-University Würzburg, 97070 Würzburg, Germany

**Keywords:** prostate cancer, PC, PET/CT, positron emission tomography, [^18^F]-PSMA, artificial learning, deep learning, DenseNet

## Abstract

**Background:** Prostate cancer (PC) is a leading cause of cancer-related deaths in men worldwide. PSMA-directed positron emission tomography (PET) has shown promising results in detecting recurrent PC and metastasis, improving the accuracy of diagnosis and treatment planning. To evaluate an artificial intelligence (AI) model based on [^18^F]-prostate specific membrane antigen (PSMA)-1007 PET datasets for the detection of local recurrence in patients with prostate cancer. **Methods:** We retrospectively analyzed 1404 [^18^F]-PSMA-1007 PET/CTs from patients with histologically confirmed prostate cancer. Artificial neural networks were trained to recognize the presence of local recurrence based on the PET data. First, the hyperparameters were optimized for an initial model (model A). Subsequently, the bladder was localized using an already published model and a model (model B) was trained only on a 20 cm cube around the bladder. Finally, two separate models were trained on the same section depending on the prostatectomy status (model C (post-prostatectomy) and model D (non-operated)). **Results:** Model A achieved an accuracy of 56% on the validation data. By restricting the region to the area around the bladder, Model B achieved a validation accuracy of 71%. When validating the specialized models according to prostatectomy status, model C achieved an accuracy of 77% and model D an accuracy of 77%. All models achieved accuracies of almost 100% on the training data, indicating overfitting. **Conclusions:** For the presented task, 1404 examinations were insufficient to reach an accuracy of over 90% even when employing data augmentation, including additional metadata and performing automated hyperparameter optimization. The low F1-score and AUC values indicate that none of the presented models produce reliable results. However, we will facilitate future research and the development of better models by openly sharing our source code and all pre-trained models for transfer learning.

## Simple Summary

Prostate cancer is a leading cause of cancer-related deaths in men around the world. A type of imaging technique called positron emission tomography (PET), which uses a special scan to detect cancer, has shown great promise in identifying recurring prostate cancer and spread to other parts of the body. In this study, we created a computer-based model that uses PET scan images to predict if prostate cancer has come back after treatment. To improve the model’s performance, we tried different methods, such as focusing on different parts of the image, adding extra information from the patient’s medical history, and including details about whether the patient had prior surgery to remove the prostate. These efforts led to an accuracy of 77% in predicting cancer recurrence. While this accuracy was lower than the desired 90%, the model still showed significant improvement. Many approaches were tested, each helping to improve the model. The results of this study are an important step forward in developing tools that can reliably detect cancer recurrence in the prostate area. However, more research is required to further improve the model’s accuracy and make it more useful for doctors in real-life situations.

## 1. Introduction

Prostate cancer remains one of the leading causes of cancer-related deaths in men, with over 1.2 million new cases diagnosed and more than 350,000 deaths annually [1,2]. Early detection and accurate staging are critical for successful treatment, and therapy effectiveness is typically monitored by tracking serum prostate-specific antigen (PSA) levels. When PSA levels rise, indicating biochemical recurrence, patients are often referred for re-staging, a process that is crucial for determining the most appropriate treatment strategy. A key focus during re-staging is identifying whether there is a local recurrence of cancer in the prostate region. Several diagnostic methods are used for staging and detecting prostate cancer, including digital rectal examination, transrectal ultrasound (TRUS), magnetic resonance imaging (MRI), and positron emission tomography (PET) combined with computed tomography (CT) [3]. Among these, PET/CT has emerged as a reliable technique [4]. In ^18^F-PSMA-PET, the radioactive tracer ^18^F-PSMA-1007 binds selectively to the prostate-specific membrane antigen (PSMA), a protein highly expressed on prostate cancer cells, and it is performed in combination with the CT scan, allowing for precise anatomical imaging. Typically, a whole-body PET/CT scan is conducted to detect potential metastases. Specifically, for prostate cancer diagnosis and staging during remission, each PET/CT examination generates high-dimensional data, comprising hundreds of image slices with thousands of pixels per slice for both PET and CT parts. Interpreting these complex data to answer the critical binary question, i.e., whether there is a local recurrence of prostate cancer in the prostate bed or not, requires trained experts.

Recently, artificial neural networks have shown great potential in assisting with similar tasks, such as tumor segmentation and outcome prediction in head and neck cancers [5], as well as the identification of pathological mediastinal lymph nodes in lung cancer [6]. These advancements highlight the promise of AI in improving diagnostic accuracy and efficiency in prostate cancer staging and treatment [7]. In parallel, the field of theranostics has emerged, promising improvements in therapy as well as diagnostics in oncology [8,9]. Together with the predictive power of novel AI methods, these developments have the potential to dramatically improve early diagnosis, prognostic staging, and patient management.

Recently, deep neural networks have successfully been trained for survival prediction of prostate cancer patients based on magnetic resonance imaging [10] and segmentation and classification of uptake patterns on ^18^F-PSMA-PET scans [11]. Therefore, the aim of this work was to develop a neural network capable of detecting local recurrence in the prostate or prostate bed with a high accuracy of at least 90% using ^18^F-PSMA-1007 PET/CT examinations. This goal of 90% accuracy was somewhat arbitrarily selected as the minimum performance to justify further exploration. For direct clinical application, even higher accuracy is required.

## 2. Materials and Methods

### 2.1. Study Population

Details concerning the study group are shown in Table 1. Briefly, 1145 patients with 1404 ^18^F-PSMA-1007 PET/CT scans performed at the Department for Nuclear Medicine at the University Hospital Würzburg between 2019 and 2023 were included in this study. The PET/CT scans were split into training (1016 scans), validation (188 scans), and test (200 scans) sets.

We used hybrid PET/CT scanners with an extended field-of-view for the PET and a 64- or 128-slice spiral CT (Biograph64 or 128, Siemens Healthineers; Erlangen, Germany). All cases were re-evaluated explicitly for this project by a trained nuclear medicine physician (H.E. using syngo.via, V60A, Siemens Healthineers; Erlangen, Germany) to label each examination with 0 (no local recurrence in the prostate region, n = 637), 1 (local recurrence in the prostate region, n = 704), or 2 (uncertain whether there is local recurrence in the prostate region). Instances with label 2 were excluded from training. In summary, the following metadata were available for each examination in addition to PET/CT scans: patient pseudo-ID, age, sex, staging, prostatectomy status, PSA level, and label.

Even though the question of local recurrence makes sense only at re-staging, we decided to include primary staging patients in the training as they provided examples of scans with cancerous tissue in the prostate region. We assigned all primary staging scans to the training set. The remaining examinations were assigned randomly into training and validation sets, ensuring that all scans from the same patient ended up in the same set.

The test set comprises 200 scans from 198 patients on the same scanners. This set contains re-staging examinations only. However, 83 patients (84 scans) already had scans in the training/validation set, while 115 patients (116 scans) were novel. Even for known patients, these are new examinations, and both metadata and the label might have changed. The parameters for measurement were identical to those from the training/validation set; the same metadata were supplied, and the same labeling procedure was employed.

### 2.2. Data Processing

**Figure 1:**
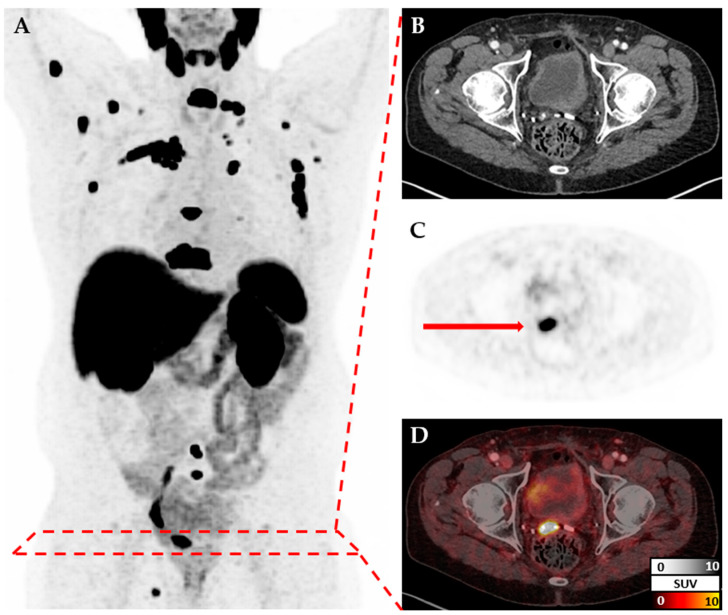
PSMA-PET/CT: (**A**) maximum intensity projection; axial corresponding CT (**B**) and PET (**C**) slices; (**D**) fused PET and CT slices. Example of a 78-year-old patient with osseus and lymphnodal metastases, as well as a local recurrence in the prostate bed (indicated by the red arrow).

Images were exported from the clinic PACS in DICOM format and converted to NIfTI via dcm2niix [12]. These NIfTIs contain intensities in Becquerels/milliliter (BQML) in order to convert them to standardized uptake calue (SUV), and scan-specific scaling factors were calculated from DICOM metadata. Data loaders for deep learning in Python (version 3.12.3) were implemented using nibabel (version 5.2.1, [13]), PyTorch (version 2.3.0, [14]), and Monai (version 1.3.1, [15]). CT and PET images for each patient were measured at different resolutions but co-registered to a common space to ensure accurate spatial alignment (Figure 1). Therefore, after reading both files individually, they were re-scaled to a matrix size of 150 × 150 × 150. Depending on the pixel spacing and slice thickness of the original scans, the resulting voxel size differed between patients. Intensity was re-scaled to a range of 0 to 1 based on the minimum and maximum values in each scan. Models with consistent scaling across scans using a fixed linear transformation were explored (Appendix A). Additional transformations like cropping or augmentation transformations were applied depending on the model. Metadata and labels were provided as a simple csv file and loaded via Pandas (version 2.2.2, [16,17]).

### 2.3. Models

As part of this project, many models were trained to explore the effects of including different kinds of data, pre-processing, and deep learning techniques. New models build on previous successful models. In addition to the initial experimentation [18], more than twenty models and variants were trained systematically. Here, we only focus on four models that show incremental improvements. All other models and their variants are described in Appendix A. Source code and trained models are shared in the GitHub repository (version 0.3.0) and on Zenodo. All models use the DenseNet architecture [19] with 121 layers, as implemented in Monai [15]. All models used cross-entropy as the loss function, the Adam optimizer, and a learning rate of 10^−5^ for 15 epochs. The batch size was set to 16, except for Model A, where this exceeded the GPU memory; in this case, a batch size of 8 was used. After each epoch, the validation accuracy was logged, and the weights were saved. We report the maximum accuracy and provide the model weights from the epoch that reached this maximum. Training was performed on an NVIDIA GeForce RTX 4090 GPU with 24 GB of RAM (Nvidia Corporation, Santa Clara CA, USA).

#### 2.3.1. Model A: Base Model

The initial model provided the baseline performance, with the most naïve approach, passing the resized data to a DenseNet121, including moderate augmentation (random rotations by up to 0.2 radians).

#### 2.3.2. Model B: Cropped Around the Prostate

**Figure 2:**
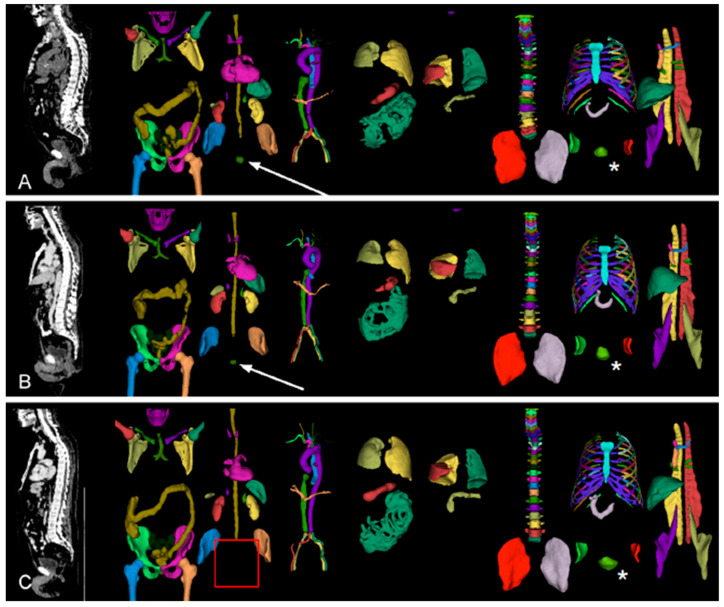
Result of TotalSegmentator [20] on three patients in Subfigures (**A**), (**B**), and (**C**). White arrows indicate the detected prostate gland in patients A and B, while the red rectangle indicates the absence of the prostate gland in patient C. The white asterisks (*) indicate that the urinary bladder was successfully detected in all patients. Color indicates the organ that was detected by TotalSegmentator.

The data include the whole body, while the specific question (local recurrence) can be answered by focusing on the area around the prostate (region). To automatically crop image volumes around the prostate, the location of the prostate was determined by TotalSegmentator (version 2.1.0, [20]). This worked in most cases, even for patients after radical prostatectomy. In the remaining cases, the position of the urinary bladder was used as a proxy (Figure 2). Images were cropped to a 20 cm × 20 cm × 20 cm cube around the centroid of the prostate (or urinary bladder) in patient coordinates and re-scaled to 70 × 70 × 70 voxels. Variants with stronger cropping were explored (Appendix A).

#### 2.3.3. Model C: Adding Prostatectomy Status and PSA Level

The status of prostatectomy (px, 0 = no prostatectomy; 1= radical prostatectomy) and the PSA levels are potentially informative. Therefore, this information was added to the model as separate image layers with repeated values (0 or 1 for px and a floating-point number with the normalized PSA level). Before training model C with integrated px information, we trained separate models for px = 0 and px = 1 (Model 5a, 5b in Appendix A). Those models had high accuracy but low balanced accuracy as they overfit the respective majority class (Appendix A).

#### 2.3.4. Model D: More Extensive Augmentation and Hyperparameter Optimization

Training usually converged rather quickly. So, to increase data heterogeneity (without generating new training sets), we employed more extensive augmentation. With a growing number of augmentation transformations, the number of hyperparameters to tune also increased. Therefore, we applied the hyperparameter optimization toolkit Optuna (version 3.6.0) [21] to determine optimal hyperparameter combinations for spatial cropping around the center (CenterSpatialCropd), random flipping (RandFlipd), and random zooming (RandZoomd).

### 2.4. Evaluation

The classification performance of all models was evaluated using the accuracy and the balanced accuracy of the validation set. Accuracy is the number of correct predictions divided by the number of total predictions. This metric is susceptible to class imbalance [22]. Therefore, this metric is complemented by the balanced accuracy, which is equivalent to the arithmetic mean of sensitivity and specificity in the case of binary classification.

Only the best-performing model was evaluated on the hold-out test set. Missing values were imputed before prediction in the few cases with missing metadata (two cases missing px; eleven cases missing PSA). For px, the majority class (px = 1), and PSA, mean values (30.1) were used. In addition to accuracy and balanced accuracy, the confusion matrix is reported. Furthermore, accuracy is separately determined for known patients and novel patients.

## 3. Results

**Figure 3:**
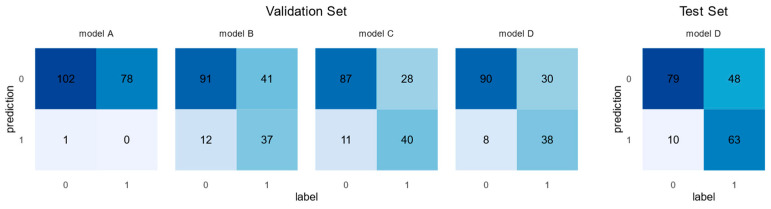
Confusion matrices of models A–D on the validation set (**left**) and model D on the test set (**right**). Columns are labels, and rows are predictions. The numbers within the cells are additionally color coded with a gradient from light blue (small numbers) to dark blue (large numbers).

The initial model A, which used the entire volume with moderate augmentation, reached an accuracy of 56.4% with a balanced accuracy of 49.5% but an F1-score of 0.0, indicating a complete lack of true positive predictions (Table 2, Figure 3). Restricting the input data to a 70 × 70 × 70 volume around the prostate gland, model B reached an accuracy of 70.7% and a balanced accuracy of 67.9%. Including prostatectomy status and PSA levels as additional data layers (model C) further increased the accuracy to 76.5% with a balanced accuracy of 73.8%. Finally, using Optuna to optimize the hyperparameters and augmentation settings returned the following settings: CenterSpatialCropd with dimensions 65 × 46 × 69, RandFlipd along the spatial axis 1 with a probability of 1 (100%), and RandZoomd with a minimum and maximum zoom of 0.5 and a probability of 1 (100%). Model D trained with these settings had the highest accuracy (77.1%) and balanced accuracy (73.9%). Neither of these models came close to our target accuracy of 90%. As the model with the highest validation accuracy (Table 2), we selected model D for evaluation on the hold-out test set.

Model D reached an accuracy of 71.0% and a balanced accuracy of 72.8% on the test set (Figure 3). While the model had high specificity (88.8%), the sensitivity was poor (56.8%). In 116 cases, the patients were not previously known. Of these cases, 74.1% were correctly identified, while 66.7% of the 84 cases with previously known patients were correctly identified.

## 4. Discussion

This work aimed to develop a highly accurate neural network to predict local recurrence in prostate cancer patients from PSMA-PET/CT images. We iteratively refined our approach as the initial naïve model had insufficient accuracy with no true positives. Specifically, we cropped the images around the prostate gland to focus on this region, included important metadata as separate image layers, and performed hyperparameter optimization of certain augmentations to increase the data heterogeneity (Table 1). Furthermore, we modified the dimensions of the region of interest and attempted to mask the PET channel in certain organs or everywhere except the prostate to let the model focus on the prostate alone (Appendix A). Interestingly, some refinements, like using a consistent intensity scaling based on SUV units across all patients (Appendix A.2.11), decreased the classification accuracy rather than improving it. This indicates that our models were unable to learn the expected proper features from the data. While most of our refinements generally increased the accuracy, the final test set accuracy of 71.0% is still far from acceptable in a clinical context. In particular, the poor sensitivity is concerning in this context as it corresponds to patients with local recurrence not being identified. Sensitivity can generally be increased using a weighted loss function or adapting the threshold for the predicted probability. However, this usually trades specificity for sensitivity. As the overall performance of our models was far from ideal, we did not attempt fine-tuning this trade-off. Furthermore, attempts to use Grad-CAM [23] to determine which regions within the volume of interest contribute most to the decision of the model returned ambiguous results. This indicates that the features that were learned are not the medically relevant features of the image. While we cannot postulate that we reached a theoretical accuracy limit for this task (given our data set), we employed many advanced techniques to improve our models iteratively and reached a comparable accuracy to the 71.4% previously reported for the prediction of prostate cancer recurrence based on ^18^F-FACBC PET [24]. By documenting this evolution and publishing all code and models, we provide a blueprint for approaching DL to answer a medical research question. While it is undoubtedly possible to marginally increase the accuracy of artificial neural networks by extending the hyperparameter space (e.g., by exploring different network architectures), we postulate that to reach accuracies above 90%, a completely different technique, sophisticated domain-specific adaptations (e.g., improved attenuation correction [25,26,27,28]), or much more data are required. Transfer learning is an interesting technique that lowers the necessary training data [29,30]. A pre-trained model on a related task is required to perform transfer learning. Alternatively, a large dataset for a related task can be used to pre-train a model yourself. Even though transfer learning has already been successfully applied for prostate cancer detection on biopsy images [31] and ultrasound [32], we could not find any suitable model or dataset, but now we provide our models as starting points for transfer learning by others. As datasets can usually not be shared because of data protection requirements, we hope our models will be used for TL and that the resulting models will be shared again. We plan to evaluate the performance of an iteratively re-trained model on the initial dataset.

## 5. Conclusions

For the presented task, 1404 examinations were insufficient to reach an accuracy of over 90% even when employing data augmentation, including additional metadata and performing automated hyperparameter optimization. The low F1-score and AUC values indicate that none of the presented models produce reliable results. However, we will facilitate future research and the development of better models by openly sharing our source code and all pre-trained models for transfer learning.

## Figures and Tables

**Table 1:** Study group characteristics.

	Total	Training	Validation	Test
Patient number	1145	868	161	198
^18^F-PSMA-PET/CT scan number	1404	1016	188	200
**Indication for ^18^F-PSMA-PET/CT scan**				
Primary staging	212	212	0	0
Re-staging	1192	804	188	200
**Patients’ characteristics**				
Age, mean (range)	70.5 (44–90)	70.3 (44–90)	70.9 (46–89)	71.3 (53–86)
Scans with prior prostatectomy (%)	796 (57%)	546 (54%)	127 (68%)	123 (62%)
PSA-level, mean (range)	44.9 (0–7434)	46.3 (0–3420)	53.8 (0–7434)	30.1 (0–932)
**Label**				
0 (no local recurrence)	637	445	103	89
1 (local recurrence)	704	515	78	111
2 (uncertain case)	63	56	7	0

**Table 2:** Model accuracies, balanced accuracies, F1-scores, and area under the receiver operating characteristic curve (AUC) on the validation set.

Model	Accuracy	Balanced Accuracy	F1-Score	AUC
Model A: Base Model	0.564	0.495	0.000	0.500
Model B: Cropped FOV	0.707	0.679	0.583	0.697
Model C: px and PSA	0.765	0.738	0.672	0.736
Model D: hyperparam.	0.771	0.739	0.667	0.753

**Figure A1:**
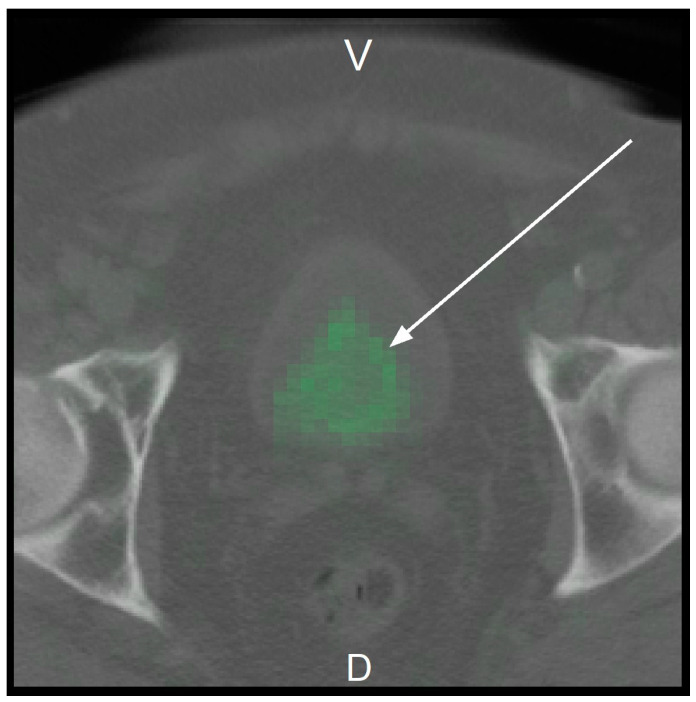
Cropped NIfTI of pseudo ID 00045.

**Figure A2:**
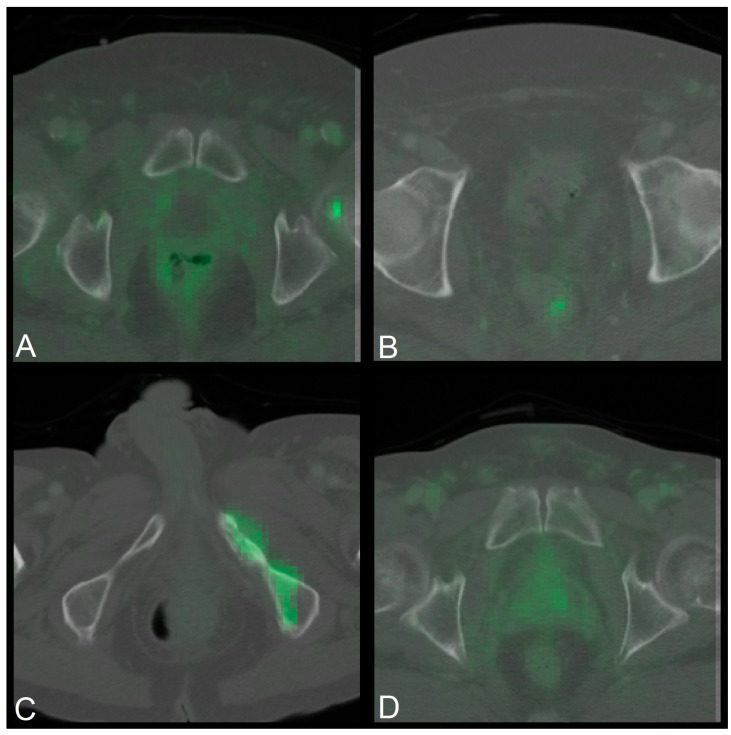
NIfTI examples that might show reasons why the model makes mistakes.

**Figure A3:**
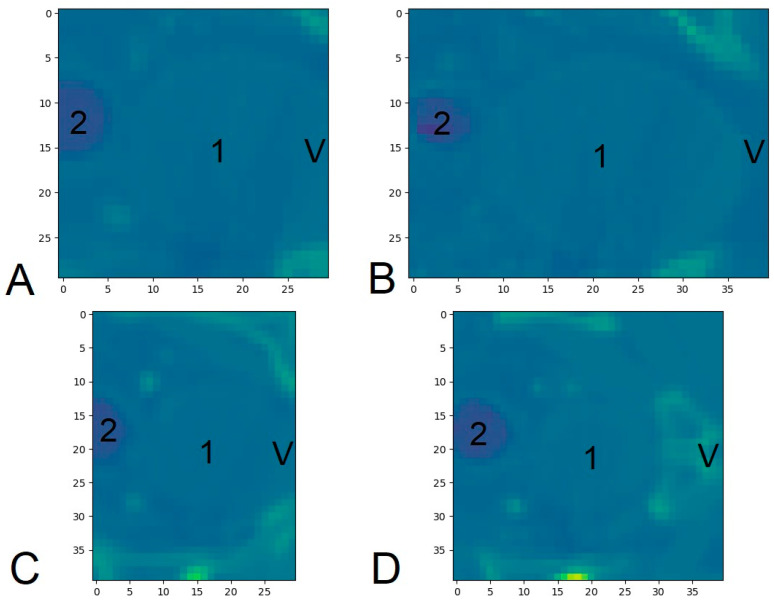
Various ROI modifications.

**Figure A4:**
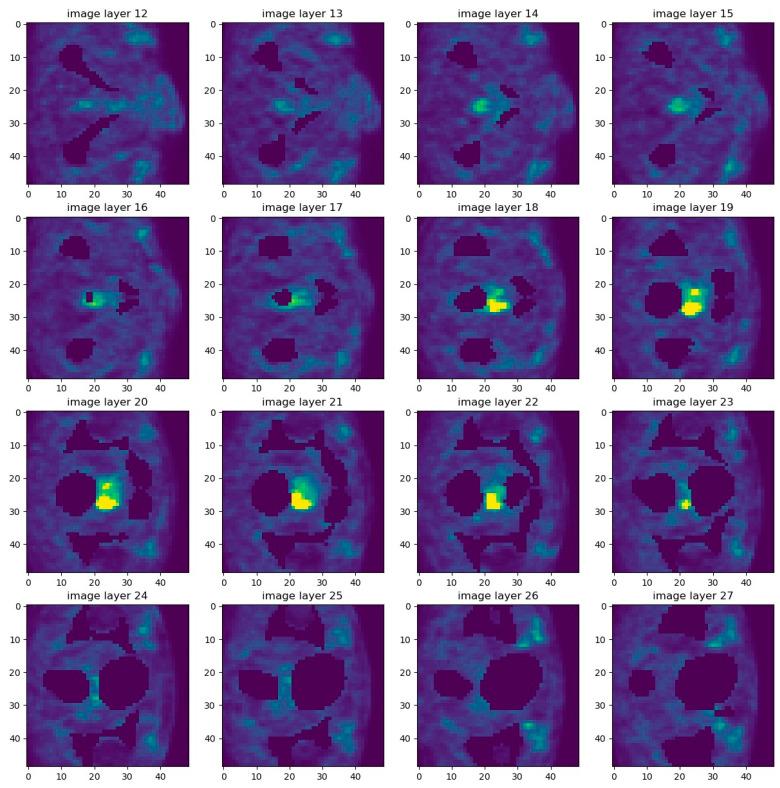
16 layers of an image with visible PET channel and masking of UB, colon and hip bones.

**Figure A5:**
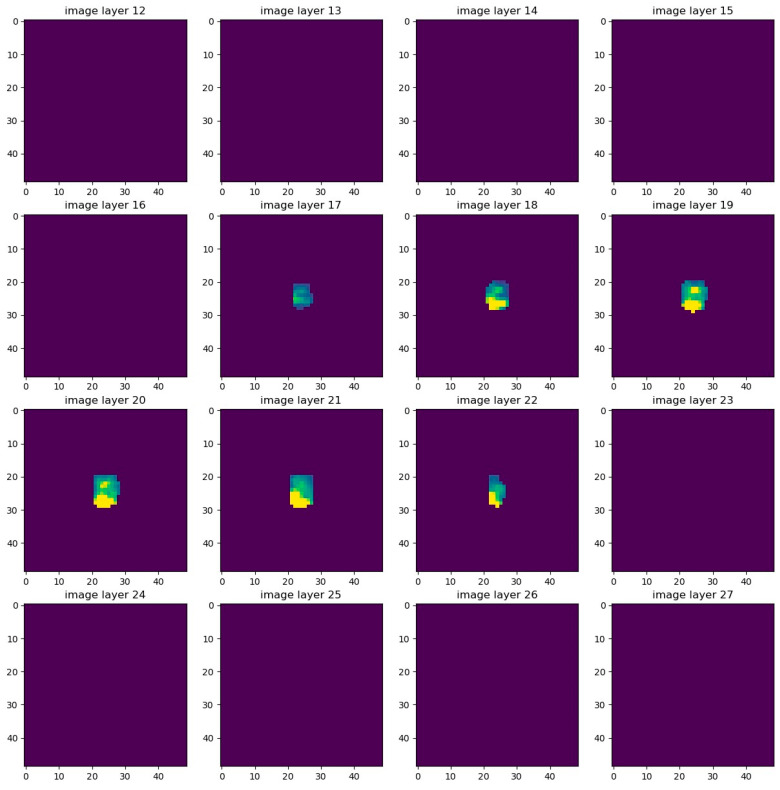
16 layers of an image with visible PET channel and masking of everything except the prostate.

## Data Availability

The datasets presented in this article are not readily available because of data protection regulations for routine clinical data. Requests to access the datasets should be directed to WS. All source code is available at https://github.com/BioMeDS/f18-psma-pet-ct-ai (accessed on 2 May 2025) and archived at Zenodo https://doi.org/10.5281/zenodo.14944344. The pre-trained model weights are deposited on Zenodo https://doi.org/10.5281/zenodo.14944879.

## Appendix A

Detailed description and evaluation of all models, including those not presented in the main text.

**AUC**	Area under the Receiver Operating Characteristic Curve
**BN**	batch normalization
**BQML**	Becquerels/milliliter
**CNN**	convolutional neural network
**Conv**	convolution
**CT**	computed tomography
**DenseNet**	Dense Convolutional Network
**MONAI**	Medical Open Network for Artificial Intelligence
**MRI**	magnetic resonance imaging
**PC**	prostate cancer
**PET**	positron emission tomography
**PSA**	prostate-specific antigen
**PSMA**	prostate-specific membrane antigen
**px**	prostatectomy state
**ReLU**	rectified linear unit
**ROI**	region of interest
**SUV**	Standardized Uptake Value
**UB**	urinary bladder
Note: this appendix contains material adapted from the master thesis of Marko Korb.	

### Appendix A.1. Material

The dataset that was used to train our models is described in the main text of the manuscript (in particular in Table 1).

Many different packages were used to write and train the model. The most important packages are in the following Table A1.

**Table A1:** Most important packages that were used.

Name	Version	Source
Jupyterlab	4.2.1	https://jupyter.org/
Mamba	1.5.8	https://mamba.readthedocs.io/en/latest/
Matplotlib	3.8.4	https://matplotlib.org/
Monai	1.3.1	https://monai.io/
Napari	0.4.19	https://napari.org/stable/
Nibabel	5.2.1	https://nipy.org/nibabel/
Numpy	1.26.4	https://numpy.org/
Optuna	3.6.0	https://optuna.org/
Pandas	2.2.2	https://pandas.pydata.org/
Python	3.12.3	https://www.python.org/
PyTorch	2.3.0	https://pytorch.org/
TotalSegmentator	2.1.0	https://totalsegmentator.com/

The models were trained on a Computer at the Center for Computational and Theoretical Biology Wu¨rzburg with a Nvidia GeForce RTX 4090 as GPU.

The different models and their explanations are listed in the following Table A2.

**Table A2:** Different Models and their explanations.

Model	Explanation
Model 1	Uncropped NIfTIs
Model 2	NIfTIs cropped around prostate
Model 3	Modification of ROI to dimensions (40, 40, 40) around prostate
Model 4a	Model 3 and masking of colon, hip bones and urinary bladder
Model 4b	Model 3 and masking of everything except prostate
Model 5a	Model 3 and separation by px = 0
Model 5b	Model 3 and separation by px = 1
Model 6a	Model 3 and px information as additional layer
Model 6b	Model 3 and psa value as additional layer
Model 6c	Model 3 and px information + psa value as additional layers
Model 7a	Optuna hyperparameter optimization of CenterSpatialCropd (A)
Model 7b	Optuna hyperparameter optimization of RandFlipd (B)
Model 7c	Optuna hyperparameter optimization of RandZoomd (C)
Model 7x	Optuna hyperparameter optimization of A, B and C
Model A	Improved version of Model 1
Model B	Improved version of Model 2
Model B scaleRange	Improved version of Model 2 with consistent intensity scaling
Model C	Improved version of Model 6c
Model C scaleRange	Improved version of Model 6c with consistent intensity scaling
Model D	Improved version of Model 7d
Model D scaleRange	Improved version of Model 7d with consistent intensity scaling

During the training of each model, a checkpoint was saved after each epoch. The checkpoint with the highest validation accuracy, as well as the train loss and metrics throughout training are deposited at Zenodo (https://doi.org/10.5281/zenodo.14944879).

### Appendix A.2. Methods and Results

The basis for training and validating the models was Medical Open Network for Artificial Intelligence (MONAI) [15]. To train the model, a convolutional neural network (CNN) DenseNet with 121 layers was used as the architecture. In MONAI, a DenseNet121 is already included and can be implemented with the following command: model = monai.networks.nets.DenseNet121(). The batch size for all models was 16. An exception was made for Model 1 and A, where it was 8 and 12, respectively. The performance of the models was assessed based on training accuracy, but more importantly, validation accuracy and balanced accuracy. The balanced accuracy is calculated on the basis of the sensitivity across all classes. In the following course of this work, the term accuracy (accuracies) refers to the validation accuracy (accuracies).

#### Appendix A.2.1. Preliminary Experiments

The foundations of the model have been established before the systematic model evaluation started. This basic model was used as Model 1 (Table A2) in this work. The provided data was used to create *Labels.tsv*. The CT and PET scans were converted from their original DICOM format to NIfTIs. All pseudo IDs that have a label of 2 were also sorted out initially, as the human observer was not sure which label (0/1) should be assigned here.

#### Appendix A.2.2. Uncropped NIfTIs (Model 1)

In Model 1, uncropped NIfTIs were used. These show the patient from the thigh to the head and therefore contain a lot of information that is not part of the objective of this work. One of these uncropped NIfTIs with computed tomography (CT) channel and positron emission tomography (PET) channel can be seen in Figure 1 in the main text.

The following transforms were used for training (Listing A1) and validation (Listing A2):

**Listing A1.** Basic transform for the training of Model 1.

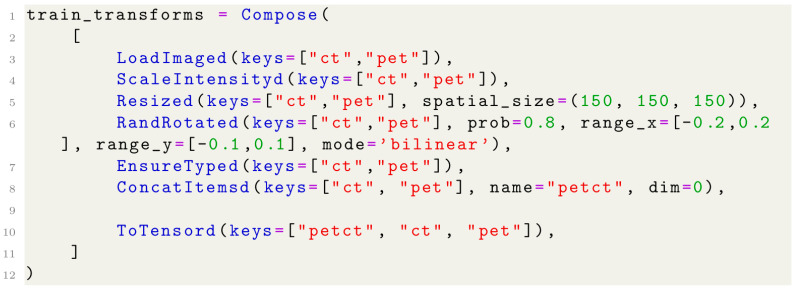

**Listing A2.** Basic transform for the validation of Model 1.

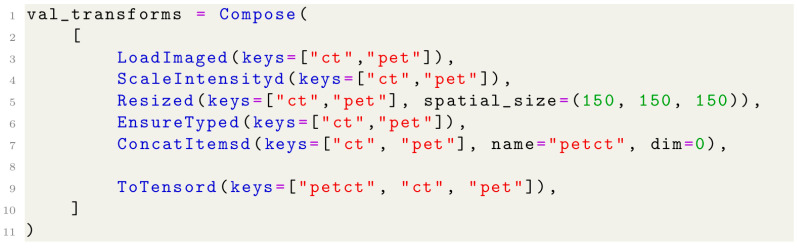

The image data and metadata are loaded with LoadImaged(keys = ["ct","pet"]). ScaleIntensityd (keys = ["ct","pet"]) scales intensity values of an image/image tensor so that the different images are easier to compare, process and visualize. To set a specific image size, Resized(keys = ["ct","pet"], spatial_size = (150, 150, 150)) is used. In this case, the image size is set to 150 x 150 x 150 voxels. To get more variation while training the model, RandRotated(keys = ["ct","pet"], prob = 0.8, range_x = [-0.2,0.2], range_y = [-0.1,0.1], mode = ’bilinear’) is used to rotate the images along the X-axis and the Y-axis. With EnsureTyped(keys = ["ct","pet"]) it is ensured that the image has the desired data type. ConcatItemsd(keys = ["ct", "pet"], name = "petct", dim = 0) merges a list of image tensors into a single image tensor. Finally, ToTensord(keys = [" petct", "ct", "pet"]) is used to convert the images into tensors.

This model achieved its best training accuracy at epoch 15 with a value of 0.7774 or 77.74% and its best validation accuracy at epoch 11 with a value of 0.6133 or 61.33%. Model 1 achieved a balanced accuracy of 0.487 or 48.7%. The results of Model 1 can be found in the following Table A3. The validation accuracy of Model 1 will be used as a reference value for the accuracy achieved by the other models.

**Table A3:** Results of Model 1.

**Model**	**Training acc.**	**Validation acc.**	**Balanced acc.**
Model 1	0.7774	0.6133	0.487

#### Appendix A.2.3. Cropping of NIfTIs Using TotalSegmentator

In order to limit the image area of the NIfTIs to the area relevant to the research question, inner body regions were first segmented using the TotalSegmentator [20]. An overview of the regions segmented by the TotalSegmentator in three different patients can be seen in Figure 2 of the main text.

Because there were 39 patients in whom no prostate (region) could be found, the cropping of the NIfTIs had to be performed in two steps. First, the NIfTIs for the cases in which the prostate (region) was detected were cropped based on the prostate. For this the segmented prostate was used as the center of the image and the new image area was defined as a cube around it. Second, in cases where no prostate (region) could be detected, the urinary bladder (UB) (Figure 2 in the main text, *) was chosen as the center of the new image area, as it is in close proximity to the prostate.

One challenge when cropping the images was to get the CT and PET images to the same image size, as they are saved at different scales. For this purpose, the start and end points of the CT and PET images were defined and calculated so that both images have the same size. A function was written for this purpose, which can be found in the following Listing A3.

**Listing A3.** Function to scale the CT and PET images to the same size.

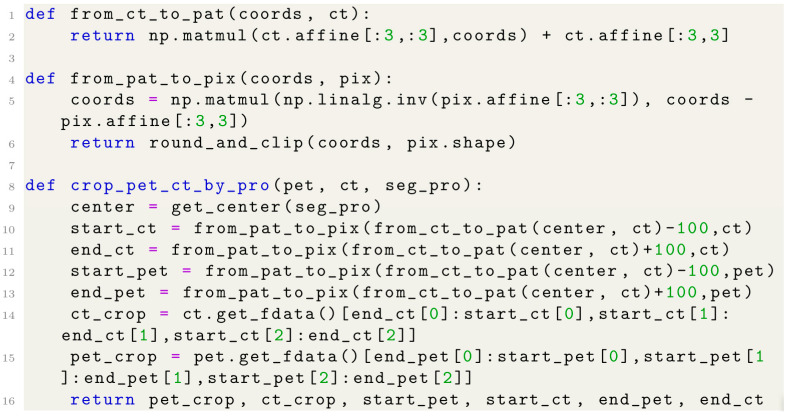

#### Appendix A.2.4. Cropped NIfTIs (Model 2)

The previously cropped NIfTIs were then used in the training of Model 2. The cropped image area can be seen as a CT image with PET image overlay in the following Figure A1.

Cropped NIfTI of pseudo ID 00045 with one channel as CT (greyscale) and one channel as PET (green). The ventral side of the patient (V) is on the top, while the dorsal side of the patient (D) is at the bottom of the Figure. The right hip bone is on the left side of the image, while the left hip bone is on the right side of the image. The Figure is in perspective, as if one were looking towards the head of the patient. The arrow points to the prostate (area).

As in Model 1, after sorting out the incorrect NIfTIs and the cases in which the label is 3, the data set had a size of 1138. Similarly, the size of the training set was 957 and the size of the validation set was 181. The training transforms and the validation transforms were identical to those in Model 1, with one difference: in function Resized(), instead of a spatial_size of (150, 150, 150), a spatial_size of (70, 70, 70) was used to enable the batch size to be increased from 8 to 16.

Model 2 achieved a training accuracy of 0.9112 or 91.12% at epoch 15, a validation accuracy of 0.7072 or 70.72% at epoch 14 and a balanced accuracy of 0.669 or 66.9%. The results of Model 2 can also be found in the following Table A4 where they are compared to the results of Model 1.

**Table A4:** Results of Model 2 in comparison to Model 1.

Model	Training acc.	Validation acc.	Balanced acc.
Model 1	0.7774	0.6133	0.487
Model 2	0.9112	0.7072	0.669

If the achieved accuracies of Model 2 are compared with those of Model 1, it can be observed that cropping to the image area relevant to the research question enabled an increase in validation accuracy of approximately 9 percentage points and in balanced accuracy of around 18 percentage points. Based on this result, the cropped NIfTIs were also used for the following models.

#### Appendix A.2.5. Manual Inspection of PET/CT Scans

Approximately 100 PET/CT images were inspected manually using the software Napari [33] to determine which features the model struggles with and what else could be done to improve the accuracy of the model. It was noted that region of interest (ROI) could be further restricted around the prostate (area) and that PET signals are also very common in the UB, hip bones and colon. The PET signals occurring in the colon in particular were often similar in size to those visible in the prostate in the case of confirmed local recurrence. Another challenge for the model could be that the prostate and UB are in close proximity which leads to the PET signal of the prostate and UB flowing into each other. A few examples can be seen in the following Figure A2.

Four NIfTIs that show different potential reasons for errors made by the model. PET signal present in contents of the colon at different positions and sizes (A)(B), hip bones showing PET signal (C), and a smooth transition of the PET signal of the prostate (area) and the PET signal of the UB (D). As in Figure A1, the ventral side of the patients is at the top of the images, while the dorsal side of the patients is at the bottom. The CT layer of the images is in greyscale, while the PET layer is shown in green.

Based on these observations, the decision was made to try modifying the ROI and masking certain areas in order to potentially improve the accuracy of the model.

#### Appendix A.2.6. Region of Interest Modification (Model 3)

To modify the ROI, the MONAI function CenterSpatialCropd(keys=["ct", "pet"], roi_size=(x, y, z)) was used in the training transforms and the validation transforms, where x, y and z represent the respective sizes from the center of the image. Otherwise, the training transforms and validation transforms are identical to those in Model 2. The dataset had a size of 1138, while the size of the training set was 957 and the size of the validation set was 181.

Four different cropped image dimensions were tried out: (30, 30, 30), (30, 40, 30), (40, 30, 40) and (40, 40, 40). An overview of these four image dimensions can be seen in the following Figure A3.

Overview of four different ROI modifications. Crop around the center of the image with a size of 30, 30, 30 (A), with a size of 30, 40, 30 (B), with a size of 40, 30, 40 (C) and a size of 40, 40, 40 (D). The ventral side of the patient was labeled V, while the UB was labeled 1 and the colon was labeled 2.

The submodel of Model 3 that uses CenterSpatialCropd(keys = ["ct", "pet"], roi_size = (30, 30, 30)) has a training accuracy of 0.8694 or 86.94% in epoch 15, a validation accuracy of 0.6906 or 69.06% in epoch 11 and a balanced accuracy of 0.677 or 67.7%.

The submodel that uses CenterSpatialCropd(keys = ["ct", "pet"], roi_size = (30, 40, 30)) has a training accuracy of 0.8652 or 86.52% in epoch 15, a validation accuracy of 0.7072 or 70.72% in epoch 15 and a balanced accuracy of 0.687 or 68.7%. The submodel that uses CenterSpatialCropd(keys = ["ct", "pet"], roi_size = (40, 30, 40)) has a training accuracy of 0.8736 or 87.36% in epoch 15, a validation accuracy of 0.7072 or 70.72% in epoch 12 and a balanced accuracy of 0.675 or 67.5%. The fourth submodel, which uses CenterSpatialCropd(keys = ["ct", "pet"], roi_size = (40, 40, 40 )) has a training accuracy of 0.8736 or 87.36% in epoch 15, a validation accuracy of 0.7072 or 70.72% in epoch 6 and a balanced accuracy of 0.627 or 62.7%. These results can be compared to the results of the previous models in the following Table A5.

**Table A5:** Results of Model 3 in comparison to the previous models.

Model	Training acc.	Validation acc.	Balanced acc.
Model 1	0.7774	0.6133	0.487
Model 2	0.9112	0.7072	0.669
Model 3 (30, 30, 30)	0.8694	0.6906	0.677
Model 3 (30, 40, 30)	0.8652	0.7072	0.687
Model 3 (40, 30, 40)	0.8736	0.7072	0.675
Model 3 (40, 40, 40)	0.8736	0.7072	0.627

Comparing the different results of Model 3 with each other and with the previous models, it can be concluded that the submodel with CenterSpatialCropd(keys = ["ct", "pet"], roi_size = (30, 40, 30)) produces the highest accuracy in combination with the highest balanced accuracy so far. An increase of about 9 percentage points compared to Model 1 was possible. Even though there was no increase of accuracy compared to Model 2, there was a slight increase of balanced accuracy. Therefore, the dimensions used here were also used in the following models. However, due to the small increase, the following models were also carried out without modifying the ROI.

#### Appendix A.2.7. Masking of Areas (Model 4)

As mentioned in Appendix A.2.5, PET signals were also visible in other structures that could be potential causes of error for the model. As a result, two approaches were pursued in the following section: First, the UB, the colon and the two hip joints were masked. In the second approach, everything except the prostate (area) was masked. For this purpose, the PET signal in the structures recognized by the TotalSegmentator was set to 0.

To mask the areas mentioned, the location information of the prostate, UB, the colon and the hip bones provided by the TotalSegmentator had to be mapped as image data to variables. Nibabel [13] was used for this (Listing A4).

**Listing A4.** Code for mapping the segmentations of the prostate, UB, colon and the hip bones.

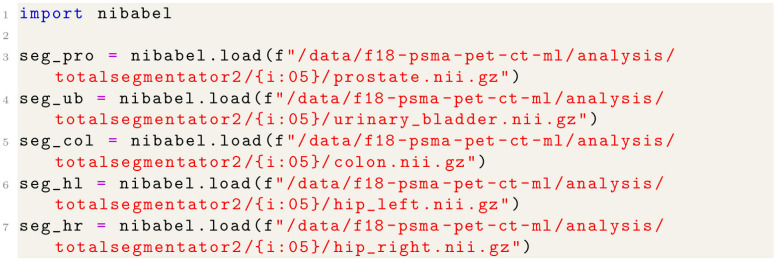

The segmentations were then resized to the size of the PET channel and the pixel values of the PET channel were set to 0 if the mask has a pixel value of 1. With this the PET signal of the UB, colon and hip bones was set to 0, masking these structures. The masked PET images were then used instead of the normal PET images for training the model.

##### Masking of Certain Structures (Model 4a)

The first approach was to mask the previously mentioned structures UB, colon and hip bones. An overview of this masking can be seen in the following Figure A4. Here, 16 image layers of the PET channel around the prostate in one patient were visualized together with the mask.

Overview of 16 image layers of the PET channel around the prostate of a patient. Between layer 18 and layer 23, the prostate can be seen as a yellow-green signal. The mask is also clearly visible, as it can be recognized as completely dark areas. The ventral side of the patient is on the right side of the images, while the dorsal side is on the left side of the images. The patient’s right hip bone is on the upper side of the images, while the patient’s left hip bone is on the lower side.

Since there were NIfTIs in which no prostate (area) could be found, the prostate was not used in these NIfTIs as the center of the masked images, but the UB. Otherwise, the prostate was used as the center of the masked images. The training transforms and the validation transforms of Model 4a were identical to those of Model 3. The dataset had a size of 1117, while the training set had a size of 939 and the validation set had a size of 178.

Model 4a achieved a training accuracy of 0.8168 or 81.68% in epoch 15, a validation accuracy of 0.6629 or 66.29% in epoch 11 and a balanced accuracy of 0.628 or 62.8%. The version of Model 4a using CenterSpatialCropd(keys=["ct", "pet"], roi_size=(30, 40, 30)) achieved a training accuracy of 0.7135 or 71.35% in epoch 12, a validation accuracy of 0.5787 or 57.87% in epoch 14 and a balanced accuracy of 0.536 or 53.6%. These results can be compared to the results of the previous models in the following Table A6.

**Table A6:** Results of Model 4a in comparison to the previous models.

Model	Training acc.	Validation acc.	Balanced acc.
Model 1	0.7774	0.6133	0.487
Model 2	0.9112	0.7072	0.669
Model 3 (30, 30, 30)	0.8694	0.6906	0.677
**Model 3 (30, 40, 30)**	**0.8652**	**0.7072**	**0.687**
Model 3 (40, 30, 40)	0.8736	0.7072	0.675
Model 3 (40, 40, 40)	0.8736	0.7072	0.627
Model 4a	0.8168	0.6629	0.628
Model 4a (30, 40, 30)	0.7135	0.5787	0.536

When comparing the results of Model 4a with each other and with the results of the previous models, it can be seen that Model 4a without CenterSpatialCropd (keys = ["ct", "pet"], roi_size = (30, 40, 30)) achieved a better training and validation accuracy. However, the accuracies of Model 4a were lower than those of Model 3 with CenterSpatialCropd(keys = ["ct", "pet"], roi_size = (30, 40, 30)). Masking the UB, the colon and the hip bones therefore does not appear to have a positive effect on the accuracy of the model. The balanced accuracies of both versions of Model 4a were lower than those of Model 3, except the version of Model 3 with CenterSpatialCropd(keys = ["ct", "pet"], roi_size = (40, 40, 40)).

##### Masking of Everything Except Prostate (Model 4b)

In the second approach, everything except the prostate was masked. So, in contrast to before, when the PET signal was removed from certain anatomical structures, in this approach, the signal is removed from the whole volume, except for the prostate (bed). An overview of this masking can be seen in the following Figure A5. Here, 16 image layers of the PET channel around the prostate in the same patient as in Figure A4 were visualized together with the mask.

Overview of 16 image layers of the PET channel around the prostate of a patient. Between layer 17 and layer 22, the prostate can be seen as a yellow-green signal, while the rest of the image is masked. The ventral side of the patient is on the right side of the images, while the dorsal side is on the left side of the images. The patient’s right side is on the upper side of the images, while the patient’s left side is on the lower side of the images.

In this approach, only data in which a prostate (area) was recognized by the TotalSegmentator was used. The training and validation transforms were again identical to those in Model 3 and Model 4a. The dataset consisted of 1081 cases, while the training set contained 909 cases and the validation set contained 172 cases.

Model 4b achieved a training accuracy of 0.8317 or 83.17% in epoch 15, a validation accuracy of 0.6047 or 60.47% in epoch 8 and a balanced accuracy of 0.554 or 55.4%. The version of Model 4b with CenterSpatialCropd (keys = ["ct", "pet"], roi_size = (30, 40, 30)) achieved a training accuracy of 0.7404 or 74.04% in epoch 15, a validation accuracy of 0.5726 or 57.26% in epoch 12 and a balanced accuracy of 0.563 or 56.3%. These results can be seen in the following Table A7 and compared with the results of the previous models.

**Table A7:** Results of Model 4b in comparison to the previous models.

Model	Training acc.	Validation acc.	Balanced acc.
Model 1	0.7774	0.6133	0.487
Model 2	0.9112	0.7072	0.669
Model 3 (30, 30, 30)	0.8694	0.6906	0.677
Model 3 (30, 40, 30)	0.8652	0.7072	0.687
Model 3 (40, 30, 40)	0.8736	0.7072	0.675
Model 3 (40, 40, 40)	0.8736	0.7072	0.627
Model 4a	0.8168	0.6629	0.628
Model 4a (30, 40, 30)	0.7135	0.5787	0.536
Model 4b	0.8317	0.6047	0.554
Model 4b (30, 40, 30)	0.7404	0.5756	0.563

A comparison of the results of Model 4b with each other and with the results of the previous models shows that the accuracies achieved here were also lower than those of Model 3. The accuracies achieved by Model 4b were also lower than those of Model 4a. Only the validation accuracy of Model 4b with CenterSpatialCropd (keys = ["ct", "pet"], roi_size = (30, 40, 30)) was equal to the validation accuracy achieved by Model 4a with CenterSpatialCropd (keys = ["ct", "pet"], roi_size = (30, 4 0, 30)). Both balanced accuracies achieved by the two versions of Model 4b were lower than those of all of those of Model 3. Also in that case, it seems that masking, especially masking everything except the prostate, does not lead to an improvement in the accuracy of the model.

#### Appendix A.2.8. Separation by Prostatectomy State (Model 5)

In Model 5, two separate models were created that analyzed the data separately according to the prostatectomy state (px). These models, as well as all models following, did not use the masked NIfTIs that were used in Model 4a and Model 4b.

##### Cases with Prior Prostatectomy (Model 5a)

Model 5a was trained with the data in which px had the value 1, in other words with the patients who had already undergone a prostatectomy. The size of the dataset was 669, while the training set consisted of 542 cases and the validation set consisted of 127 cases. The training and validation transforms were identical to those of Model 3.

Model 5a achieved a training accuracy of 0.8007 or 80.07% in epoch 15, a validation accuracy of 0.7165 or 71.65% in epoch 4 and a balanced accuracy of 0.484 or 48.4%. The version of Model 5a that used CenterSpatialCropd(keys = ["ct", "pet"], roi_size = (30, 40, 30)) achieved a training accuracy of 0.8395 or 83.95% in epoch 15, a validation accuracy of 0.7165 or 71.65% in epoch 8 and a balanced accuracy of 0.496 or 49.6%. These results are shown in the following Table A8 were they can also be compared to the results of the previous models.

**Table A8:** Results of Model 5a in comparison to the previous models.

Model	Training acc.	Validation acc.	Balanced acc.
Model 1	0.7774	0.6133	0.487
Model 2	0.9112	0.7072	0.669
Model 3 (30, 30, 30)	0.8694	0.6906	0.677
Model 3 (30, 40, 30)	0.8652	0.7072	0.687
Model 3 (40, 30, 40)	0.8736	0.7072	0.675
Model 3 (40, 40, 40)	0.8736	0.7072	0.627
Model 4a	0.8168	0.6629	0.628
Model 4a (30, 40, 30)	0.7135	0.5787	0.536
Model 4b	0.8317	0.6047	0.554
Model 4b (30, 40, 30)	0.7404	0.5756	0.563
Model 5a	0.8007	0.7165	0.484
Model 5a (30, 40, 30)	0.8395	0.7165	0.496

If the results of 5a are compared with each other and with the results of the previous models, it is clear that both versions of Model 5a have achieved the same validation accuracy. Even when compared with the previous best model, Model 3, an almost identical validation accuracy was achieved. The balanced accuracies achieved are, however, around 20 percentage points lower than those of Model 3 and are similar to the achieved balanced accuracy of Model 1.

##### Cases with No Prior Prostatectomy (Model 5b)

In Model 5b, the data were analyzed for px with a value of 0, meaning patients who had not yet had a prostatectomy. The dataset had a size of 469, while the training set had a size of 415 and the validation set had a size of 54. The training and validation transforms were identical to those of the previous models.

Model 5b achieved a training accuracy of 0.9325 or 93.25% in epoch 15, a validation accuracy of 0.7963 or 79.63% in epoch 1 and a balanced accuracy of 0.5 or 50%.

The version of Model 5b with CenterSpatialCropd(keys = ["ct", "pet"], roi_size = (30,40, 30)) achieved a training accuracy of 0.8454 or 84.54% in epoch 14, a validation accuracy of 0.7963 or 79.63% in epoch 4 and a balanced accuracy of 0.488 or 48.8%. In the following Table A9 the results of Model 5a can be seen and compared to the results of all previous models.

**Table A9:** Results of Model 5b in comparison to the previous models.

Model	Training acc.	Validation acc.	Balanced acc.
Model 1	0.7774	0.6133	0.487
Model 2	0.9112	0.7072	0.669
Model 3 (30, 30, 30)	0.8694	0.6906	0.677
Model 3 (30, 40, 30)	0.8652	0.7072	0.687
Model 3 (40, 30, 40)	0.8736	0.7072	0.675
Model 3 (40, 40, 40)	0.8736	0.7072	0.627
Model 4a	0.8168	0.6629	0.628
Model 4a (30, 40, 30)	0.7135	0.5787	0.536
Model 4b	0.8317	0.6047	0.554
Model 4b (30, 40, 30)	0.7404	0.5756	0.563
Model 5a	0.8007	0.7165	0.484
Model 5a (30, 40, 30)	0.8395	0.7165	0.496
Model 5b	0.9325	0.7963	0.5
Model 5b (30, 40 30)	0.9518	0.7963	0.488

Observing Table A9, it shows that Model 5b has achieved the highest validation accuracy so far. Both versions of Model 5b have achieved an identical accuracy. In this case, the accuracy of 79.63% is about 8 percentage points higher than that of Model 3 and Model 5a. This higher accuracy could, however, also be attributed to the smaller validation set. On one hand, it seems that splitting the data by px, especially with a value of 0 for px, has a positive impact on the accuracy of the model. On the other hand, the balanced accuracies of both versions of Model 5b are very low. In the case of the regular version of Model 5b, the model predicted a 1 for all pseudo IDs, which is why the model was correct in 50% of cases and wrong in 50% of cases, resulting in the balanced accuracy of 0.5 or 50%. Therefore it can be concluded that Model 5b has not really learned to recognize a local recurrence, but has merely guessed.

#### Appendix A.2.9. Additional Layers (Model 6)

In order to make more extensive use of certain metadata from the *Labels.tsv* file, it was considered to add these as additional layers to the images. The px information and the normalized prostate-specific antigen (PSA) value were selected as suitable metadata.

##### Prostatectomy State as Additional Layer (Model 6a)

In Model 6a the px information was used as an additional layer. For this purpose, a function was written that reads in the metadata and repeats it over the entire image area. The code of the function can be found in the following listing A5.

**Listing A5.** Function that reads in metadata and repeats it over the entire image area.

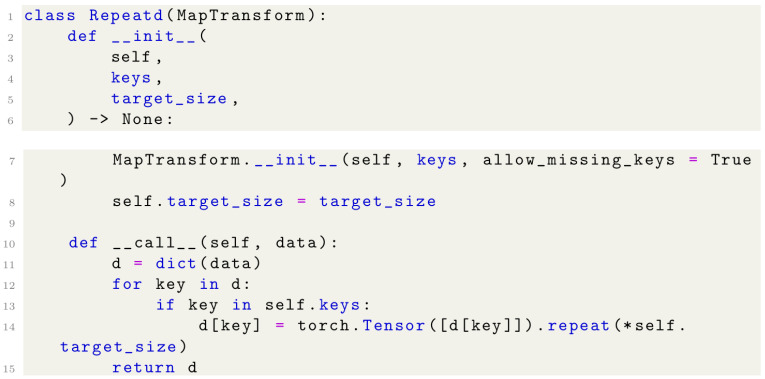

The function had to be included in the training and validation transforms of the model. This was done using Repeatd (keys = ["px"], target_size = (1, x, y, z)),, where x, y and z represent the respective sizes of the image dimensions. The complete dataset had a size of 1138 and was split into a training set of size 957 and a validation set of size 181. It was important to change the in channels to 3 when creating the model, because it now not only used ”ct” and ”pet” as input channels, but also ”px”.

Model 6a achieved a training accuracy of 0.8192 or 81.92% in epoch 10, a validation accuracy of 0.7514 or 75.14% in epoch 13 and a balanced accuracy of 0.712 or 71.2%. The version of Model 6a with CenterSpatialCropd (keys = ["ct", "pet"], roi_size = (30, 40, 30)) achieved a training accuracy of 0.8454 or 84.54% in epoch 14, a validation accuracy of 0.7514 or 75.14% in epoch 1 and a balanced accuracy of 0.698 or 69.8%. The results can be found in the following Table A10, where they can be compared to the results of the previous models.

**Table A10:** Results of Model 6a in comparison to the previous models.

Model	Training acc.	Validation acc.	Balanced acc.
Model 1	0.7774	0.6133	0.487
Model 2	0.9112	0.7072	0.669
Model 3 (30, 30, 30)	0.8694	0.6906	0.677
Model 3 (30, 40, 30)	0.8652	0.7072	0.687
Model 3 (40, 30, 40)	0.8736	0.7072	0.675
Model 3 (40, 40, 40)	0.8736	0.7072	0.627
Model 4a	0.8168	0.6629	0.628
Model 4a (30, 40, 30)	0.7135	0.5787	0.536
Model 4b	0.8317	0.6047	0.554
Model 4b (30, 40, 30)	0.7404	0.5756	0.563
Model 5a	0.8007	0.7165	0.484
Model 5a (30, 40, 30)	0.8395	0.7165	0.496
Model 5b	0.9325	0.7963	0.5
Model 5b (30, 40 30)	0.9518	0.7963	0.488
Model 6a	0.8192	0.7514	0.712
Model 6a (30, 40, 30)	0.8454	0.7514	0.698

If the results of Model 6a are compared with each other and with the results of the previous models, it becomes apparent that both versions of Model 6a have achieved an identical validation accuracy. It is higher than the accuracy achieved by Model 3, but lower than the result of Model 5b, which was trained separately with only the cases where px has a value of 0. Nevertheless, it can be said that using the px information as an additional layer in the images has a positive influence on the achieved validation accuracy of the model. Additionally, Model 6a achieved the highest balanced accuracy so far. In combination with the highest validation accuracy in a model that does not separate using the px information, Model 6a was the best performing model up to this point.

##### PSA as Additional Layer (Model 6b)

The normalized PSA value was used as an additional layer in Model 6b. Here the provided PSA values in *Labels.tsv* had to be normalized first. This was done using the code in the following Listing A6.

**Listing A6.** Normalization of the PSA values.

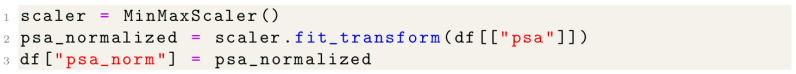

Like in Model 6a, the function in was used to read in and repeat the metadata over the whole image area. Again Repeatd(keys = ["psa"], target_size = (1, x, y, z)), had to be included in the training and validation transforms and identical to Model 6a, the in channels had to be set to 3, because Model 6b uses ”ct”, ”pet” and ”psa norm” as input channels. The dataset had a size of 1058, with a training set of size 892 and a validation set of size 166. This discrepancy to the set sizes of Model 6a is due to the fact that some cases had to be sorted out because no information on the PSA value was available for them.

Model 6b achieved a training accuracy of 0.8655 or 86.55% in epoch 15, a validation accuracy of 0.7048 or 70.48% in epoch 11 and a balanced accuracy of 0.626 or 62.6%. The version of Model 6b with CenterSpatialCropd(keys = ["ct", "pet"], roi_size = (30, 40, 30)) achieved a training accuracy of 0.8823 or 88.23% in epoch 15, a validation accuracy of 0.6627 or 66.27% in epoch 5 and a balanced accuracy of 0.646 or 64.6%. These results can be seen in the following Table A11.

**Table A11:** Results of Model 6b in comparison to the previous models.

Model	Training acc.	Validation acc.	Balanced acc.
Model 1	0.7774	0.6133	0.487
Model 2	0.9112	0.7072	0.669
Model 3 (30, 30, 30)	0.8694	0.6906	0.677
Model 3 (30, 40, 30)	0.8652	0.7072	0.687
Model 3 (40, 30, 40)	0.8736	0.7072	0.675
Model 3 (40, 40, 40)	0.8736	0.7072	0.627
Model 4a	0.8168	0.6629	0.628
Model 4a (30, 40, 30)	0.7135	0.5787	0.536
Model 4b	0.8317	0.6047	0.554
Model 4b (30, 40, 30)	0.7404	0.5756	0.563
Model 5a	0.8007	0.7165	0.484
Model 5a (30, 40, 30)	0.8395	0.7165	0.496
Model 5b	0.9325	0.7963	0.5
Model 5b (30, 40 30)	0.9518	0.7963	0.488
Model 6a	0.8192	0.7514	0.712
Model 6a (30, 40, 30)	0.8454	0.7514	0.698
Model 6b	0.8655	0.7048	0.626
Model 6b (30, 40, 30)	0.8823	0.6627	0.646

Looking at the results in Table A11, it can be seen that the version of Model 6b without CenterSpatialCropd(keys = ["ct", "pet"], roi_size = (30, 40, 30)) achieved a higher accuracy than the version that uses CenterSpatialCropd(keys = ["ct", "pet"], roi_size = (30, 40, 30)). Nevertheless, the accuracy achieved here was about 5 percentage points lower than the accuracy of Model 6a and about 9 percentage points lower than the validation accuracy of Model 5b. It seems that including the normalized PSA value as an additional image layer does not positively influence the accuracy of the model. This is also reflected in the balanced accuracies achieved, as these are lower than those of Model 6a. Despite this, in a submodel of Model 6 (Model 6c), the normalized PSA value was included together with the px information as an additional image layers.

##### Prostatectomy State and PSA as Additional Layers (Model 6c)

In Model 6c, both the px information and the normalized PSA value were used as additional image layers for training the model. As with Model 6b, it was important to normalize the PSA values in *Labels.tsv* in advance. This was also done with the code in Listing A6. The in channels had to be set to 4, as Model 6c uses ”ct”, ”pet”, ”px” and ”psa norm” as input channels. The dataset had a size of 1058, while the training set had a size of 892 and the validation set had a size of 166. Again, the sizes of the sets can be explained by the fact that some entries in *Labels.tsv* did not have values for PSA.

Model 6c achieved a training accuracy of 0.8117 or 81.17% in epoch 10, a validation accuracy of 0.7590 or 75.90% in epoch 13 and a balanced accuracy of 0.723 or 72.3%. The version of Model 6c with CenterSpatialCropd (keys = ["ct", "pet"], roi_size = (30, 40, 30)) achieved a training accuracy of 0.8217 or 82.17% in epoch 14, a validation accuracy of 0.7590 or 75.90% in epoch 1 and a balanced accuracy of 0.713 or 72.3%. In the following Table A12 these results can be seen and compared to the results of the previous models.

**Table A12:** Results of Model 6c in comparison to the previous models.

Model	Training acc.	Validation acc.	Balanced acc.
Model 1	0.7774	0.6133	0.487
Model 2	0.9112	0.7072	0.669
Model 3 (30, 30, 30)	0.8694	0.6906	0.677
Model 3 (30, 40, 30)	0.8652	0.7072	0.687
Model 3 (40, 30, 40)	0.8736	0.7072	0.675
Model 3 (40, 40, 40)	0.8736	0.7072	0.627
Model 4a	0.8168	0.6629	0.628
Model 4a (30, 40, 30)	0.7135	0.5787	0.536
Model 4b	0.8317	0.6047	0.554
Model 4b (30, 40, 30)	0.7404	0.5756	0.563
Model 5a	0.8007	0.7165	0.484
Model 5a (30, 40, 30)	0.8395	0.7165	0.496
Model 5b	0.9325	0.7963	0.5
Model 5b (30, 40 30)	0.9518	0.7963	0.488
Model 6a	0.8192	0.7514	0.712
Model 6a (30, 40, 30)	0.8454	0.7514	0.698
Model 6b	0.8655	0.7048	0.626
Model 6b (30, 40, 30)	0.8823	0.6627	0.646
**Model 6c**	**0.8117**	**0.7590**	**0.723**
Model 6c (30, 40, 30)	0.8217	0.7590	0.713

When the results of model 6c are considered and compared, it can be seen that they are identical in both versions and that essentially the same level of accuracy as in model 6a was achieved. Compared to Model 6b, the increase in accuracy by approximately 5 percentage points is most likely due to the inclusion of the px information as an additional image layer. Although the accuracies achieved by Model 6a and 6c are almost identical, the px information and the normalized PSA value were used for training in Models 7a–7d, since the inclusion of the normalized PSA value in combination with the px information did not lead to a decrease in the accuracy of the model, compared to model 6a, which only used the px information as an additional layer. In addition, there was a minimal increase in balanced accuracy compared to Model 6a.

#### Appendix A.2.10. Hyperparameter Optimization Using Optuna (Model 7)

With the help of Optuna [21], a hyperparameter optimization of certain hyperparameters was carried out. These hyperparameters were the x, y and z values of CenterSpatialCropd, the probability of augmentation of the MONAI transforms RandFlipd and RandZoomd, and the minimum and maximum zoom of RandZoomd. The transform RandFlipd flips the image along a chosen axis, while RandZoomd zooms in or out of the image. Model 6c was used as the basis for the hyperparameter optimization, as it achieved the best validation accuracy so far without filtering the data according to the px information. The basic transforms for the hyperparameter optimization can be found in the following Listing A7.

**Listing A7.** Basic transforms used in the hyperparameter optimization.

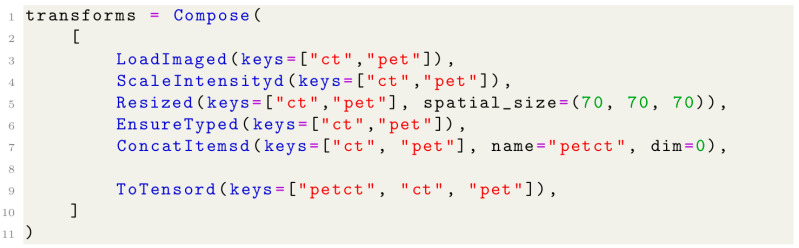

Since the px information and the normalized PSA value were used as additional layers and, as already mentioned, there are some entries in *Labels.tsv* that have no value for PSA, the data set had a size of 1058, the training set a size of 892 and a validation set with a size of 166. To optimize the hyperparameters, Optuna was given the following flexibility for certain parameters: The probability that the augmentations (RandFlipd and RandZoomd) are applied can have a value from 0 to 1 in steps of 0.1. The x, y and z values of CenterSpatialCropd can each have a value between 30 and 70. The minimum and maximum value for RandZoomd can be a value between 0.5 and 1.5 in steps of 0.1. The code for this can be seen in Listing A8.

**Listing A8.** Optuna has a defined scope for certain parameters.

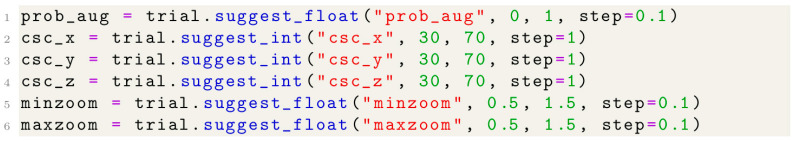

Line 1 of Listing A8 is the probability that augmentation will be applied. Lines 2 to 4 determine the x, y and z values for CenterSpatialCropd. In lines 5 and 6 the minimum and maximum values for the zoom of the MONAI transform RandZoomd.

Afterwards the training and validation transforms were defined, but as these are different for each model, they will be shown for the respective models. Each hyperparameter optimization ran through 25 trials, with each trial consisting of 15 epochs. Afterwards, the hyperparameters that achieved the highest validation accuracy in the hyperparameter optimization by Optuna were used in the training of a model.

##### CenterSpatialCropd (Model 7a)

For Model 7a, Optuna was tasked with performing a hyperparameter optimization for the x, y and z values of CenterSpatialCropd. After 25 trials with 15 epochs each, Optuna achieved the best accuracy with the following values: CenterSpatialCropd( keys = ["ct", "pet"], roi_size = (57, 30, 36)). In trial 21, the hyperparameter optimization for Model 7a achieved a training accuracy of 0.8117 or 81.17% in epoch 14 and a validation accuracy of 0.7711 or 77.11% in epoch 15. Based on the chosen x, y and z values for CenterSpatialCropd the training transforms and validation transforms of Model 7a looked like this (Listings A9 and A10):

**Listing A9.** Training transforms of Model 7a.

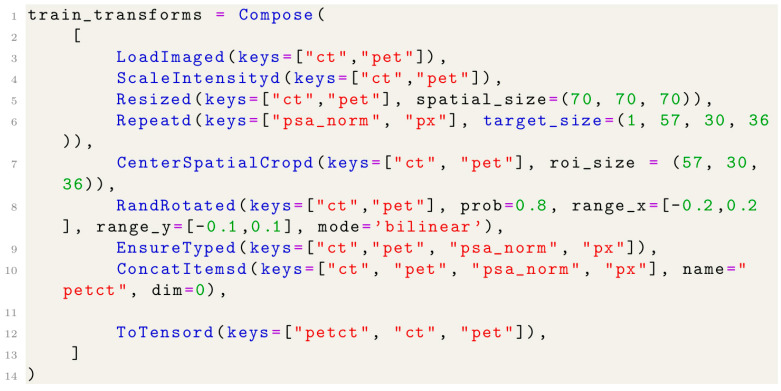

**Listing A10.** Validation transforms of Model 7a.

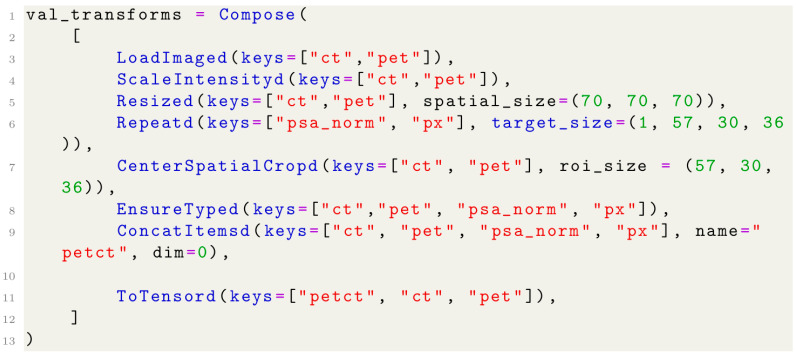

Model 7a achieved a training accuracy of 0.8251 or 82.51% in epoch 15, a validation accuracy of 0.7530 or 75.30% in epoch 1 and a balanced accuracy of 0.722 or 72.2%. The results of Model 7a can be viewed in the following Table A13 and compared with the results of the previous models.

**Table A13:** Results of Model 7a in comparison to the previous models.

Model	Training acc.	Validation acc.	Balanced acc.
Model 1	0.7774	0.6133	0.487
Model 2	0.9112	0.7072	0.669
Model 3 (30, 30, 30)	0.8694	0.6906	0.677
Model 3 (30, 40, 30)	0.8652	0.7072	0.687
Model 3 (40, 30, 40)	0.8736	0.7072	0.675
Model 3 (40, 40, 40)	0.8736	0.7072	0.627
Model 4a	0.8168	0.6629	0.628
Model 4a (30, 40, 30)	0.7135	0.5787	0.536
Model 4b	0.8317	0.6047	0.554
Model 4b (30, 40, 30)	0.7404	0.5756	0.563
Model 5a	0.8007	0.7165	0.484
Model 5a (30, 40, 30)	0.8395	0.7165	0.496
Model 5b	0.9325	0.7963	0.5
Model 5b (30, 40 30)	0.9518	0.7963	0.488
Model 6a	0.8192	0.7514	0.712
Model 6a (30, 40, 30)	0.8454	0.7514	0.698
Model 6b	0.8655	0.7048	0.626
Model 6b (30, 40, 30)	0.8823	0.6627	0.646
Model 6c	0.8117	0.7590	0.723
Model 6c (30, 40, 30)	0.8217	0.7590	0.713
Model 7a	0.8251	0.7530	0.722

Looking at Table A13, it can be seen that Model 7a achieved almost the same validation accuracy as Model 6c. If the result is compared in particular with the results of Model 3, in which CenterSpatialCropd was used and different x, y and z values were tested, it becomes clear that the values that Optuna selected provided significantly better accuracy. An improvement in validation accuracy of around 5 to 6 percentage points was possible. This can also be seen, if the achieved balanced accuracies are compared with each other. Model 7a achieved an almost identical balanced accuracy as Model 6c. However, the similarity makes it seem as if the different x, y and z values for CenterSpatialCropd do not have much effect on the accuracy of the model. The increased accuracy compared to that of Model 3 can probably be attributed to the inclusion of the px information and the normalized PSA value as additional layers.

##### RandFlipd (Model 7b)

For Model 7b, a hyperparameter optimization for the probability of using the MONAI transform RandFlipd was performed. After 25 trials with 15 epochs each, Optuna achieved the best validation accuracy with RandFlipd(keys = ["ct", "pet"], prob = 0.0 , spatial_axis = 1), which means that images were flipped with a probability of 0 based on one axis. In the first trial the model achieved a training accuracy of 0.7993 or 79.93% in epoch 1 and a validation accuracy of 0.7590 or 75.90% in epoch 15. After the hyperparameter optimization the training transforms and the validation transforms of Model 7b looked like this (Listings A11 and A12):

**Listing A11.** Training transforms of Model 7b.

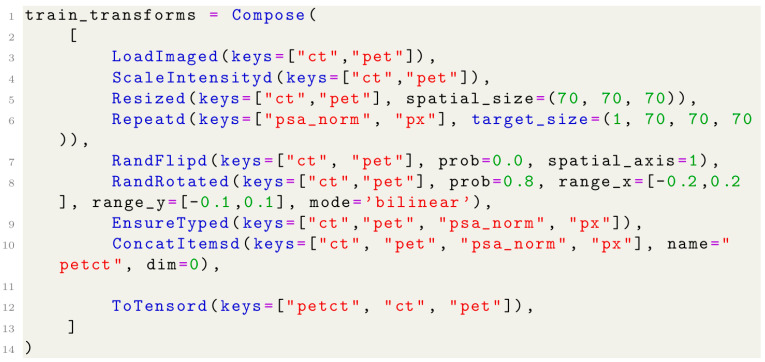

**Listing A12.** Validation transforms of Model 7b.

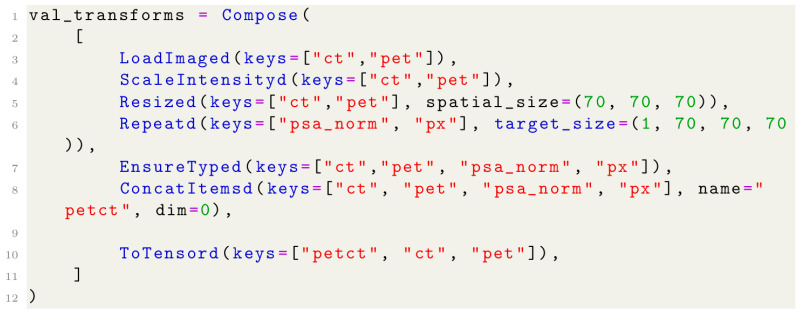

Model 7b achieved a training accuracy of 0.8150 or 81.50% in epoch 13, a validation accuracy of 0.7590 or 75.90% in epoch 1 and a balanced accuracy of 0.726 or 72.6%. The results of Model 7b can be seen in the following Table A14.

**Table A14:** Results of Model 7b in comparison to the previous models.

Model	Training acc.	Validation acc.	Balanced acc.
Model 1	0.7774	0.6133	0.487
Model 2	0.9112	0.7072	0.669
Model 3 (30, 30, 30)	0.8694	0.6906	0.677
Model 3 (30, 40, 30)	0.8652	0.7072	0.687
Model 3 (40, 30, 40)	0.8736	0.7072	0.675
Model 3 (40, 40, 40)	0.8736	0.7072	0.627
Model 4a	0.8168	0.6629	0.628
Model 4a (30, 40, 30)	0.7135	0.5787	0.536
Model 4b	0.8317	0.6047	0.554
Model 4b (30, 40, 30)	0.7404	0.5756	0.563
Model 5a	0.8007	0.7165	0.484
Model 5a (30, 40, 30)	0.8395	0.7165	0.496
Model 5b	0.9325	0.7963	0.5
Model 5b (30, 40 30)	0.9518	0.7963	0.488
Model 6a	0.8192	0.7514	0.712
Model 6a (30, 40, 30)	0.8454	0.7514	0.698
Model 6b	0.8655	0.7048	0.626
Model 6b (30, 40, 30)	0.8823	0.6627	0.646
Model 6c	0.8117	0.7590	0.723
Model 6c (30, 40, 30)	0.8217	0.7590	0.713
Model 7a	0.8251	0.7530	0.722
Model 7b	0.8150	0.7590	0.726

Observing the results, visible in Table A14, it can be seen that the same accuracy and balanced accuracy were achieved for Model 7b as for Model 6c. An explanation for this could be the fact that no augmentation took place due to the value 0.0 for the probability of augmentation. Model 7b therefore shows no difference to Model 6c, on which it is based.

##### RandZoomd (Model 7c)

A hyperparameter optimization for the MONAI transform RandZoomd was carried out for Model 7c. The probability of augmentation and the minimum and maximum value of the zoom of Optuna were selected here. The best accuracy was achieved with the parameters RandZoomd(keys = ["ct", "pet"], prob = 0.7, min_zoom = 0.5, max_zoom = 1.5 ) in trial 2 with a training accuracy of 0.8004 or 80.04% in epoch 13 and a validation accuracy of 0.7590 or 75.90% in epoch 8. Based on the hyperparameter optimization the training transforms and validation transforms of Model 7c looked like this (Listings A13 and A14):

**Listing A13.** Training transforms of Model 7c.

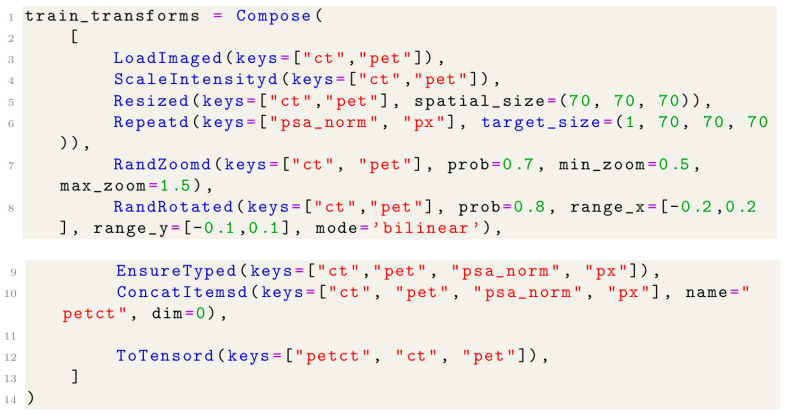

**Listing A14.** Validation transforms of Model 7c.

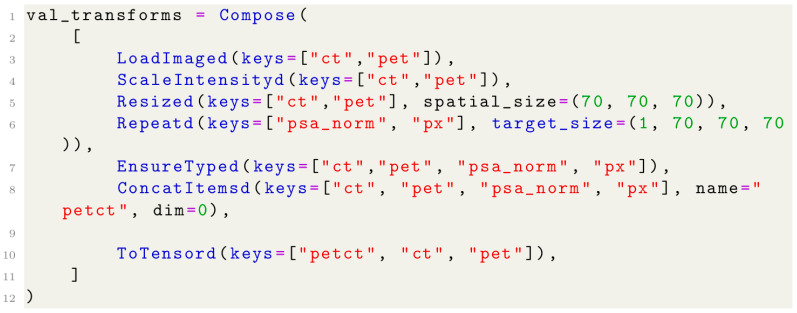

Model 7c achieved a training accuracy of 0.8027 or 80.27% in epoch 8, a validation accuracy of 0.7590 or 75.90% in epoch 13 and a balanced accuracy of 0.708 or 70.8%. The results of Model 7c can be seen and compared to the results of the previous models in the following Table A15.

**Table A15:** Results of Model 7c in comparison to the previous models.

Model	Training acc.	Validation acc.	Balanced acc.
Model 1	0.7774	0.6133	0.487
Model 2	0.9112	0.7072	0.669
Model 3 (30, 30, 30)	0.8694	0.6906	0.677
Model 3 (30, 40, 30)	0.8652	0.7072	0.687
Model 3 (40, 30, 40)	0.8736	0.7072	0.675
Model 3 (40, 40, 40)	0.8736	0.7072	0.627
Model 4a	0.8168	0.6629	0.628
Model 4a (30, 40, 30)	0.7135	0.5787	0.536
Model 4b	0.8317	0.6047	0.554
Model 4b (30, 40, 30)	0.7404	0.5756	0.563
Model 5a	0.8007	0.7165	0.484
Model 5a (30, 40, 30)	0.8395	0.7165	0.496
Model 5b	0.9325	0.7963	0.5
Model 5b (30, 40 30)	0.9518	0.7963	0.488
Model 6a	0.8192	0.7514	0.712
Model 6a (30, 40, 30)	0.8454	0.7514	0.698
Model 6b	0.8655	0.7048	0.626
Model 6b (30, 40, 30)	0.8823	0.6627	0.646
Model 6c	0.8117	0.7590	0.723
Model 6c (30, 40, 30)	0.8217	0.7590	0.713
Model 7a	0.8251	0.7530	0.722
Model 7b	0.8150	0.7590	0.726
Model 7c	0.8027	0.7590	0.708

Model 7c achieved the same validation accuracy as Models 7a, 7b and 6c, although in this case augmentation was applied with a probability of 70%. Based on this, it can be assumed that zooming in and out of the image has no further positive effect on the validation accuracy. The balanced accuracy was slightly lower than in the 4 previous models.

##### CenterSpatialCropd + RandFlipd + RandZoomd (Model 7d)

Finally, the previously used transforms were combined for Model 7d. Here, a hyperparameter optimization was performed for CenterSpatialCropd, RandFlipd and RandZoomd. With CenterSpatialCropd(keys = ["ct", "pet"], roi_size = (65, 46, 69)), RandFlipd(keys = ["ct", "pet"], prob = 1.0, spatial_axis = 1) and RandZoomd(keys = ["ct","pet"], prob = 1.0, min_zoom = 0.5, max_zoom = 0.5), the Model 7d had the best accuracy. In trial 7 the model achieved a training accuracy of 0.8016 or 80.16% in epoch 5 and a validation accuracy of 0.7590 or 75.90% in epoch 4. Based on the chosen hyperparameters, the training transforms and validation transforms of Model 7a looked like this (Listings A15 and A16):

**Listing A15.** Training transforms of Model 7d.

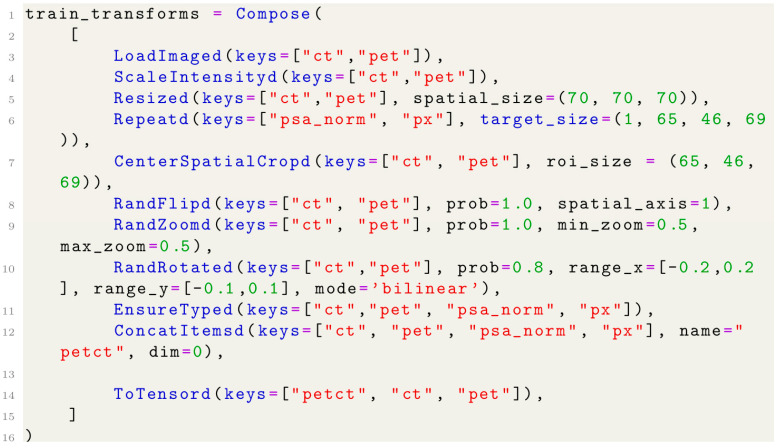

**Listing A16.** Validation transforms of Model 7d.

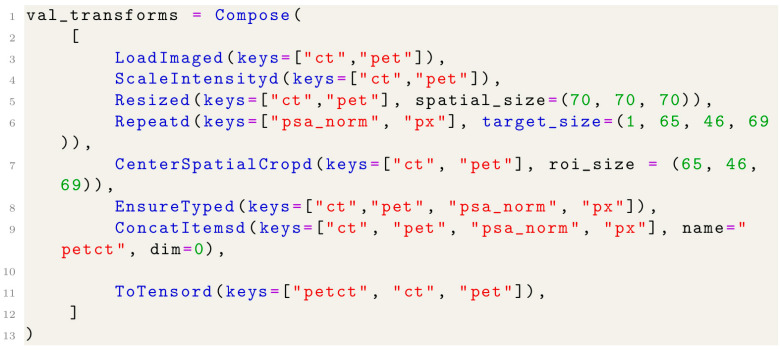

Model 7d achieved a training accuracy of 0.8184 or 81.84% in epoch 12, a validation accuracy of 0.7711 or 77.11% in epoch 10 and a balanced accuracy of 0.706 or 70.6%. The results of Model 7d can be viewed in the following Table A16 and compared with the results of the previous models.

**Table A16:** Results of Model 7d in comparison to the previous models.

Model	Training acc.	Validation acc.	Balanced acc.
Model 1	0.7774	0.6133	0.487
Model 2	0.9112	0.7072	0.669
Model 3 (30, 30, 30)	0.8694	0.6906	0.677
Model 3 (30, 40, 30)	0.8652	0.7072	0.687
Model 3 (40, 30, 40)	0.8736	0.7072	0.675
Model 3 (40, 40, 40)	0.8736	0.7072	0.627
Model 4a	0.8168	0.6629	0.628
Model 4a (30, 40, 30)	0.7135	0.5787	0.536
Model 4b	0.8317	0.6047	0.554
Model 4b (30, 40, 30)	0.7404	0.5756	0.563
Model 5a	0.8007	0.7165	0.484
Model 5a (30, 40, 30)	0.8395	0.7165	0.496
Model 5b	0.9325	0.7963	0.5
Model 5b (30, 40 30)	0.9518	0.7963	0.488
Model 6a	0.8192	0.7514	0.712
Model 6a (30, 40, 30)	0.8454	0.7514	0.698
Model 6b	0.8655	0.7048	0.626
Model 6b (30, 40, 30)	0.8823	0.6627	0.646
Model 6c	0.8117	0.7590	0.723
Model 6c (30, 40, 30)	0.8217	0.7590	0.713
Model 7a	0.8251	0.7530	0.722
Model 7b	0.8150	0.7590	0.726
Model 7c	0.8027	0.7590	0.708
Model 7d	0.8184	0.7711	0.706

Looking at Table A16, it can be seen that Model 7d achieved the highest validation accuracy up to this point. Comparing this result with the previous models, it could be assumed that the two MONAI transforms RandFlipd and RandZoomd in connection with CenterSpatialCropd lead to a slight increase in accuracy. While the valdiation accuracy increased, the balanced accuracy slighty decreased. It is almost identical to the balanced accuracy of Model 7c but lower than in the Models 7a, 7b and 6c. In addition, it seems that the validation accuracy of 0.7590 or 75.90% is like a hard limit that the model can only overcome with difficulty.

#### Appendix A.2.11. Models in the Main Text

For the main text of the manuscript, initially models 1, 2, 6c and 7d were selected. Based on feedback from the reviewers, the data transformation and code were refined to use intensity in SUV rather than BQML units, consistently interpolate CT and PET with the bilinear method in the rotation augmentation and streamline the code (e.g. drop EnsureChannelFirst). The resulting models are now called model A to D. Models B to D were trained again using consistent scaling of the intensity values using ScaleIntensityRanged rather than ScaleIntensityd using values derived from the intensity distribution in the cube arount the prostate across the training and validation set. See Listing A17.

**Listing A17.** Consistent intensity scaling used in variants of models B–D.

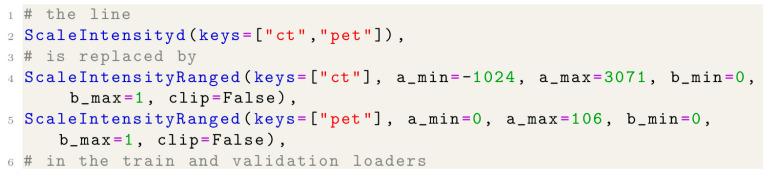

These models reached the following accuracies (Table A17).

**Table A17:** Results of models selected for the manuscript and their variants.

Model	Validation acc.	Balanced acc.	F1 Score	AUC
Model A	0.564	0.495	0.000	0.500
Model B	0.707	0.679	0.583	0.697
Model B scaleRange	0.630	0.623	0.573	0.643
Model C	0.765	0.738	0.672	0.736
Model C scaleRange	0.753	0.723	0.650	0.707
Model D	0.771	0.739	0.667	0.753
Model D scaleRange	0.759	0.731	0.661	0.753

Interestingly, the models trained with consistently scaled images perform worse than the models trained with dynamically scaled images.

### Appendix A.3. Discussion

The initial model Model 1 achieved an accuracy of 61.33% and a balanced accuracy of 48.7%, so it still had a lot of room for improvement. This poor performance can be attributed to the fact that the image data used depicted the body of the patient from the thighs to the head. Since the research question of this work deals with the prostate, or the prostate region, there was too much information in the NIfTIs that biased the model away from determining local recurrence in the prostate area. Balanced accuracy was used as an additional metric to determine the performance of the model. This is calculated on the basis of sensitivity (true positive) and specificity (true negative), which is important because false positives and false negatives have different degrees of severity in the medical context of the research question. For example, it would be less serious for the patient if PC is diagnosed even though no cancer is actually present than if existing PC is not detected.

To ensure that the model could focus better on the prostate area, the image data was segmented using the TotalSegmentator and then cropped to create a cube around the prostate or UB. The cropping alone increased the achieved accuracy to 70.72% and the balanced accuracy to 66.9%, which showed that the model was now unlikely to be distracted by areas of the image that were unimportant for the research question. However, the accuracy achieved could still have been significantly higher.

To further restrict the image area around the prostate, different x, y and z dimensions of the MONAI transform CenterSpatialCropd were used. The model that used CenterSpatialCropd(keys=["ct", "pet"], roi_size = (30, 40, 30)) achieved the best performance with an accuracy of 70.72% and a balanced accuracy of 68.7%. Further modification of the ROI did not improve the accuracy, but a small improvement in the balanced accuracy was possible. This could be due to the fact that the image area around the prostate was already sufficiently restricted or not yet perfectly restricted. Since the modification of the ROI only led to a minimal increase in balanced accuracy, the future models were run with and without modification of the ROI.

In order to further improve the performance of the model, potential sources of error were assessed by manual inspection of PET/CT images. PET signals in areas close to the prostate such as the UB, the colon or the hip bones were suspected as potential sources of error. Two approaches were tried to eliminate these signals: First, the aforementioned structures were masked at the PET level, meaning the PET value at the location of the structures was set to 0. Second, the PET values were set to 0 in the entire image area, except for the prostate. However, this did not lead to an improvement in performance, but instead caused it to drop. The highest accuracy achieved by Models 4a and 4b was 66.29%, while the highest balanced accuracy was 62.8%. Both values were achieved in model 4a without CenterSpatialCropd(keys=["ct ", "pet"], roi_size = (30, 40, 30)). The reason for this could be that the masking suppressed PET signals that are still relevant for determining a local recurrence of PC, even if they are not located directly on the prostate.

In Model 5, the data and training were separated by px. Model 5a, which dealt with the cases in which a prostatectomy had taken place, achieved a maximum accuracy of 71.65% and a maximum balanced accuracy of 49.6%. Model 5b, which trained on the cases with no previous prostatectomy, achieved a maximum accuracy of 79.53% and a maximum balanced accuracy of 50%. At first glance, the performance of the Model 5, especially the Model 5b, seems like a big improvement.

However, the balanced accuracy reveals a weakness of Model 5: the data appears to be very biased, which is why the model either only predicts 0 or 1 or only very rarely deviates from one of the two values. Unfortunately, the separation by px in the context of the data of this work is therefore not an improvement of the model but a serious downgrade. The model does not learn from the data provided, but simply guesses the label that occurs most frequently in the data set in order to achieve a better accuracy. No deep learning would have to be used for this. This case in particular shows why more than one metric should be used to evaluate the performance of a model.

To make better use of the metadata provided, the px information and the normalized PSA value were added to the image data as additional image layers. Three different models were trained: One that contains only px as an additional layer, one that contains only the normalized PSA value as an additional layer, and one that contains px and the normalized PSA value as additional layers. The highest accuracy and balanced accuracy was obtained from Model 6c (px and normalized PSA value). The accuracy here was 75.90% and the balanced accuracy 72.3%. Model 6a (only px as an additional layer) achieved similar accuracies, while Model 6b (only normalized PSA value as an additional layer) achieved a lower accuracy. It can be concluded that the inclusion of the px information as an additional layer led to an improvement in accuracy, while the inclusion of the normalized PSA value did not, but also did not lead to a deterioration in accuracy. The level of the PSA value does not appear to have any significance for the model as to whether a local recurrence is present or not. Since Model 6c had a slightly better balanced accuracy than Model 6a, the px information and the normalized PSA value were used as additional layers for the future model.

With the help of hyperparameter optimization and other augmentations, attempts were made to further increase the performance of the model. Four different models with different augmentations were trained for this purpose. Model 7d, which used the augmentations CenterSpatialCropd, RandFlipd and RandZoomd, achieved the highest accuracy with a value of 77.11% and a balanced accuracy of 70.6%. The highest balanced accuracy was achieved by Model 7b, which used augmentation RandFlipd, with a value of 72.6%. Based on these results, it can be concluded in general that the inclusion of further augmentations does not necessarily have a positive effect on the performance of a model. In the case of models 7a to 7d, however, there was also no deterioration in performance. A mixture of all the augmentations used provided the best, if only slightly better, performance here. If the validation accuracies achieved are compared with the training accuracies, it can be seen that they are relatively similar. Looking at the previous models, in particular Model 2 to Model 4b, it shows that the validation and training accuracies differ much more. It can therefore be said that the use of different augmentations reduces overfitting by artificially increasing the diversity of the data. The models without these additional augmentations are more likely to show overfitting, as the training accuracies here are around 90% and thus very high, while the validation accuracies are much lower. Here, the models have probably memorized more than in models 7a bid 7d. Model 7a shows that the best x, y and z values for CenterSpatialCropd have not yet been found in Model 3. However, it cannot be said that these were found in the course of the hyperparameter optimization, as this did not have an unlimited number of trials and epochs.

Transfer learning was an approach to train a model on other PET/CT data and then train it on our data to increase the diversity even more. Alternatively, a similar, already existing model was searched for, which could have been used as a reference model. However, the search for a similar model did not result in a suitable match, as the code was either not accessible, a completely different architecture was used or it was a model for segmentation, not classification. As a result, the search was redirected to a suitable data set. The HECKTOR Challenge dataset initially proved to be the most promising, as it fulfilled the desired characteristics. It is three-dimensional image data with PET and CT layers and metadata is available. In the case of this data, however, the focus was on head and neck tumors. When looking at the metadata, it became clear that the data set was very biased. Of 488 cases, only 96 showed a relapse, while 392 showed no relapse. As a result, this data set could not be used either. As no other data sets were found during the research period, transfer learning could not be performed.

After training the different models, Model 7d was used as the best model to determine the final performance based on the test set. Since the test set could be divided into known and unknown patients, the predictions were also performed separately for known/unknown. As already mentioned in the main text, missing values in the *labels ts2024.tsv* file were replaced by imputation. For the cases of unknown patients, the model achieved an accuracy of 74.14% and for the cases of known patients an accuracy of 62.29%. Since the model achieved a higher accuracy for the unknown patients, which is similar to that of models 6a and 7a to 7d, it can be said that the model is indeed able to achieve an accuracy of 74.14% with unfamiliar data, since it could not memorize the new data beforehand. For the known patients, however, a higher accuracy than 62.29% was expected. Although the image data here is also new, these are patients who have already contributed to the training of the model with previous images.

In conclusion, it can be said that many different methods were used to improve the performance of the model. The highest accuracy achieved where the model truly learned from the data was 77,11% in Model D. An increase of approximately 16 percentage points from 61.33% was therefore possible in the scope of this project. However, the accuracy achieved is not high enough for clinical application. For this, the accuracy should be in the range of 95% to 100%. Nevertheless, important insights were gained in the course of this work, which can serve as a basis for the (further) training of such a model, which is intended to detect local recurrence in the prostate (region). Such a model could also potentially be used to detect local recurrences in other regions of the body.
